# Performance Verification of Impact Machines for Testing Plastics

**DOI:** 10.6028/jres.104.034

**Published:** 1999-12-01

**Authors:** T. A. Siewert, D. P. Vigliotti, L. B. Dirling, C. N. McCowan

**Affiliations:** National Institute of Standards and Technology, Boulder, Colorado 80303

**Keywords:** aluminum, Charpy impact test, Izod impact test, plastics, steel, verification, V-notch

## Abstract

Valid comparison of impact test energies reported by various organizations and over time depends on consistent performance of impact test machines. This paper investigates the influence of various specimen and test parameters on impact energies in the 1 J to 2 J range for both Charpy V-notch and Izod procedures, leading toward the identification of a suitable material for use in a program to verify machine performance. We investigated the influences on the absorbed energy of machine design, test material, specimen cross sectional area, and machine energy range. For comparison to published round robin data on common plastics, this study used some common metallic alloys, including those used in the international verification program for metals impact machines and in informal calibration programs of tensile machines. The alloys that were evaluated include AISI type 4340 steel, and five aluminum alloys: 2014-T6, 2024-T351, 2219-T87, 6061-T6, and 7075-T6. We found that certain metallic alloys have coefficients of variation comparable to those of the best plastics that are reported in the literature. Also, we found that the differences in absorbed energy between two designs of machines are smaller than the differences that can be attributed to the specimens alone.

## 1. Introduction

About 40 years ago, a requirement for the use of verification specimens was added to the standard for impact testing of metals, American Society for Testing and Materials (ASTM) Standard E 23 [1]. This occurred because the metals impact testing community discovered that verification tests of impact machine performance using reference specimens were able to detect certain energy loss mechanisms, mechanisms that could not otherwise be observed during traditional physics-based measurements of machine performance (pendulum period, mass, mechanical friction, windage, etc.). This present paper evaluates the use of verification specimens for machines used to test plastics, and suggests what information these specimens provide about machine performance.

Few studies on performance issues for plastics impact machines could be found. For our purposes, one of the most useful was the one used to support the precision statement in ASTM Standard D 256, “Standard Test Methods for Impact Resistance of Plastics and Electrical Insulating Materials” [2]. That report describes a round robin that included six different plastics and 25 different laboratories. It indicates that both the materials and the laboratories make significant contributions to the uncertainty in the data. Another study, also an ASTM research report, indicates that the effect of notch radius (for plastic materials) is linear over the range of notch radii of 0.03 mm to 2.5 mm [3].

## 2. Procedure

### 2.1 Material Selection

The first material to be included in the test plan was AISI type 4340 steel (of a special, high-purity grade) since this has been used for many years to make verification specimens for metals impact machines. Therefore, it serves as a good benchmark against which other materials can be measured. We compared this 4340 steel to several aluminum alloys: 2014-T6, 2024-T351, 2219-T87, 6061-T6, and 7075-T6. Alloy 6061-T6 was selected because of its reproducible performance in some informal tensile testing programs. The other aluminum alloys were selected because they are readily available, and also because they are known to possess a good blend of strength and ductility in structural applications. All these aluminum alloys have a lower modulus (stiffness), about 70 GPa, than the 4340 steel, about 200 GPa [4]. This means that they deform at a force lower than for steels, yet still at a force much greater than for plastics, whose moduli usually fall between 2 GPa and 12 GPa [5]. A higher modulus in the verification test material is not necessarily detrimental, since it can serve to better identify an energy loss mechanism in an impact machine that is due to internal friction in the components that are loaded during fracture. The larger oscillation during these higher loads with metal specimens helps us to determine whether this effect is significant during routine testing with plastic specimens.

Stability of impact energy over time is one of the most desirable features in the verification specimens used to assess machine performance. The 4340 steel has a shelf life of at least several years, and so is a good benchmark against which other materials can be measured. Although aluminum alloys such as the 2000, 6000, and 7000 series age harden, these effects were minimized through careful selection of alloy and lots. The 6061 and 7075 alloys were treated at elevated temperature (although lower than the tempering treatments for the 4340 steel), and the steep reduction in diffusion rate with temperature drastically limits subsequent aging at room temperature. The 2000 series alloys will age at room temperature, but 80 % to 90 % of the hardening is completed in 4 to 5 days [6]. To minimize the small amount of residual aging, we took our specimens from bars and plates that had been in inventory for several years. In summary, we expect very little change over time in the mechanical properties of specimens made from these metallic materials.

Another desirable feature for verification specimens is complete fracture of the specimens during impact. Complete fracture is preferred because we can compare the marks on both fractured halves. The specimens develop marks during the initial strike (when the pendulum hits the specimen), during fracture, and during subsequent collisions as the specimens leave the machines. Assessment of these marks during the post-test evaluation of the specimens (part of the metals impact test ASTM Standard E 23 procedures) provides guidance to machine owners about alignment problems and wear.

We did not include any plastics in this evaluation, because sufficient reference data exist in the two reports cited in the introduction. The goal of this study was to compare the data for these candidate metals to the existing data for the plastics.

### 2.2 Specimen Design and Preparation

**Fig. 1:**
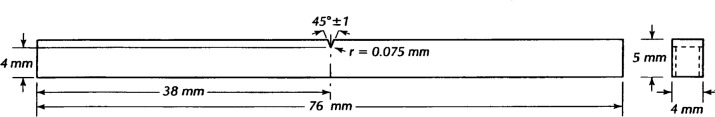
Charpy specimen design. When side grooves were used, each side was grooved to a depth of 0.25 mm with a 45° cutter.

We selected a specimen configuration (Fig. 1) that was designed to supplement the machine verification tests described in International Organization for Standardization (ISO) Draft International Standard (DIS) 13802 [7]. The dimensions were selected based on the plastic specimens described in ASTM Standard D256, but were changed where necessary to allow for the different materials properties of the metals. We permitted deviations from the standard configuration, such as side grooves on each side of the notch in some specimens, and allowing the length to be that specified either for Charpy or for Izod impact testing. One of the goals of this study was to determine the energies that would be developed by specimens of various sizes. The approach taken in the metals impact standard is to verify the machine performance, using specimens distributed over the useful range of the machine (up to 80 % of the machine capacity). For a 22 J impact machine, this means specimens with energies from 1 J or 2 J, up to about 15 J.

The standard configuration for the type 4340 steel specimens used in metal impact machines (as described in ASTM Standard E 23) has a cross section of 10 mm by 10 mm [1]. When heat-treated to produce a low energy, this specimen configuration absorbs about 15 J of energy from the pendulum. This 15 J specimen seemed appropriate to evaluate the performance of our plastics impact machine when configured for its maximum capacity of 22 J. However, our machine was damaged in an attempt to break one of these specimens in some preliminary tests. Apparently, our machine is able to tolerate energies in this range only when the fracture event is spread over a longer time, such as when the low modulus plastic specimens deform before fracture. The impact energy is a single number that is the integral of the incremental resistance of the specimen to fracture as the pendulum swings through its range. Therefore, by itself, the impact energy reading is an inaccurate way to compare the responses of low- and high-modulus materials, since it does not reflect the influence of the maximum load on the machine-specimen interactions.

After the impact machine was repaired, we continued our initial evaluations (to establish the experiment design) using miniature specimens designed to evaluate only the lower end of the machine range, between 1 J and 2 J. We selected steel specimen cross sections of 5 mm by 5 mm, 4 mm by 5 mm (notched across the 4 mm face), and 4 mm by 4 mm for some preliminary tests. This size range was designed to determine the cross section that would produce energies within the desired range, as well as a specimen size that would completely fracture upon impact. Another reason for concentrating on specimens of lower energy in the rest of this paper is that metal specimens show more ductility in thinner sections. Future tests with larger specimens, for the higher end of the machine capacity, should exhibit a more brittle fracture.

The impact data from the four specimens tested with each of the three cross sections, 4 mm by 4 mm, 4 mm by 5 mm, and 5 mm by 5 mm, were compared to the cross-sectional area. To obtain the cross-sectional area, we multiplied the two dimensions and subtracted the area of the notched region (1 mm deep) from the product. We found a linear relationship between cross-sectional area and energy (at least for this limited energy range), then used these data to standardize on a specimen dimension of 4 mm by 5 mm for the majority of our tests, since this size fell near the center of the desired energy range.

Unfortunately, the specimens in these preliminary tests did not completely separate into halves upon impact. Even the 5 mm by 5 mm specimens left the machine with the two halves still joined by a thin ligament, but bent at about a 90° angle by the impact. The ligament was so thin that the specimen halves could be bent to a 180° angle with two fingers, at which point the two halves would separate. Therefore, the specimen absorbed almost all the energy needed to fracture it (and also absorbed the kinetic (toss) energy imparted by the striker), but we did not gain data on possible jamming between the striker and anvils that might occur as broken halves left the machine. While the standard 10 mm by 10 mm specimen in a metals impact test shows almost no ductility and leaves the machine in two halves traveling at high velocity, all specimens in the range of 4 mm by 4 mm to 5 mm by 5 mm showed substantial ductility. Therefore, an optimal cross section is nearer to 10 mm by 10 mm, but we decided not to increase the section size since it would increase the energy beyond the desired range.

A thorough evaluation of all variables calls for a full factorial experimental program with a large number of replicate tests, which was beyond the scope, budget, and time available for this paper. Rather, this paper is an initial evaluation to determine which variables might be most important and should provide the basis for selection of the variables to include in a future round robin. Nevertheless, we have used statistical summaries of the data using common formulas to give some estimates of repeatability.

### 2.3 Machine Design

Our plastics impact machine can evaluate both Charpy V-notch and Izod specimens, which allowed us to develop data in the two different test configurations. The number of specimens tested using the Izod technique was much smaller than that tested using the Charpy technique, and was sufficient only to estimate if there were some differences in the coefficients of variation for these specimens between the two configurations. Although many companies follow the Charpy V-notch and Izod impact test procedures described in ASTM Standard D 256, we have seen a growing interest in ISO Standard DIS 13802, and decided to follow its procedural requirements. Differences between these two standards include anvil spacing, included angle and radius of the striker tip, anvil radius, and many other details of the test procedure. These differences preclude direct comparison of the means between specimens tested according to the two standards, but we believe that the differences are sufficiently minor to conclude that the standard deviations and coefficients of variation (CVs, or relative standard deviations) should be about the same.

While most tests were performed using a single machine set up for a full-scale capacity of 22 J, we also removed the masses bolted to the pendulum, reducing the machine range for several tests to 5 J. We also performed some tests using a conventional metals impact machine with a capacity of 358 J, and using another design of plastics impact machine configured for a capacity of 22 J.

## 3. Results and Discussion

All of the data from our tests are included in Table 1 (Charpy) and Table 2 (Izod). This body of data consists of sets of similar specimens, typically sets of 3 to 10 specimens each, so we could evaluate the scatter between the specimens tested at the same set of conditions. A summary of these data is listed in Table 3, which combines the data in each set.

Tables 1 and 2 contain columns that describe all of the parameters that were varied or measured in the evaluation. Even when summarized in Table 3, this large body of data makes comparisons difficult, so we have selected subsets of the data in the following discussion to highlight the effects of the different parameters. These subsets exclude the columns with data that were held constant (listed in the notes below each table), but add columns with the calculated standard deviations and coefficients of variation. For the 4340 steel, comparisons are made only between data from sets that were heat treated in the same batch (with similar prefixes).

### 3.1 Test Material

Table 4 includes summary data for the six materials (one steel and five aluminum alloys) included in this investigation, and is intended primarily to compare the repeatability of the different materials. The statistical data should be used with care in comparing the different materials, since the values are based on only three specimens for each material. Also, standard deviations cannot be compared fairly when the means are different. Therefore, the next column lists the coefficients of variation (CVs), which are the standard deviations divided by the means, and which are also commonly called relative standard deviations. These permit easier comparison of repeatability among specimen types and shapes with different means, but they do also suffer from the same statistical deficiency that comes from having only three specimens. We can thus make only broad generalizations about the data.

The data for the metals seem to fit into three groups according to the coefficient of variation: CV up to 0.036, CV near 0.05, and CV near 0.1. We compared these to the interlaboratory data reported for plastics in Table 1 of ASTM Standard D256-93a [2]. These data fell into two groups: coefficients of variation between 0.042 and 0.058 for the plastics with lower energy absorption (phenolic, acetal, reinforced nylon, and polypropylene), and coefficients of variation between 0.012 and 0.018 for the plastics with higher energy absorption (ABS and polycarbonate). The summary of these data in ASTM Standard D256 does not mention the material thickness; instead, it uses the usual plastics convention of normalizing the energy to 25 mm of specimen width (notch length). This facilitates comparison of plastics of different sheet thicknesses (often 3 mm to 12.7 mm), but makes analysis of the data in terms of machine energy range more difficult. Nevertheless, for a given machine range, the plastics with lower energy absorption will obviously yield data that are at the lower end of that machine range.

The ductile plastics have low CVs and so should be suitable for assessing machine repeatability at the high end of the machine range. At the low end of the machine range, the metals with the lower CVs (especially 4340 steel) beat the best of the plastics, by about a factor of two. Therefore, at the low end of the machine capacity, metals offer the possibility of at least matching the ability of specimens of plastics in resolving machine repeatability or the source of machine uncertainties. In addition, metals specimens put a larger load on the machine striker and frame, better revealing mounting and other structural problems.

### 3.2 Side Grooves

Table 5 shows that side grooves reduce the mean energy (by an amount much greater than that explained by the reduction in cross-sectional area), but the coefficient of variation either stayed about the same or increased slightly. Also, the specimen halves were still joined by a similarly sized ligament. These unexpected results do not support more than a few confirming tests on aluminum alloys in any future evaluation.

### 3.3 Notch Radius

Table 6 shows the effect of halving the notch radius from 0.5 mm to 0.25 mm. In general, notches are stress concentrators that reduce the ability of a material to sustain a load and promote brittle, rather than ductile, failure [8]. In addition, sharper notches should decrease the scatter, as a sharper notch increases the local stress at the crack, and its variable contribution to the scatter. However, these data show that the consistency was much worse with the sharper notch, although the absorbed energy indeed decreased by about 10 %. Once again, these disappointing results seem to minimize the value of including a large number of tests for this variable in any future evaluations.

### 3.4 Machine Capacity and Design

Table 7 shows the effect of machine capacity and design. There was as much as 45 % variation in the energy as the machine capacity was changed from 22 J to 358 J, at least for alloy 7075. This variation is several times greater than the standard deviation and so appears to be significant. However, the effect does not seem significant for alloy 6061-T6, with the effect being less than the standard deviation. This lack of significance is also evident for changes in the machine range from 5 J to 22 J, comparison of plastics impact machines from two different manufacturers, and comparison between the plastics impact machines (with capacities of 22 J) and a metals impact machine (with a capacity of 358 J). In retrospect, it would have been better to have made more specimens of 4340 and repeated this test using specimens having a smaller standard deviation. However, both these results support the robustness of the pendulum impact machine concept and indicate that it has been implemented consistently in these different machine designs.

### 3.5 Charpy Versus Izod

The limited data prevent us from drawing strong conclusions about the effect of test orientation (Izod versus Charpy), but an informal comparison of the two types of data summarized in Table 3 reveals no clear distinction between the two. It seems as though the difference in mean energy between Izod and Charpy tests is less than the variability due to other test parameters and so cannot be resolved. The same statement can be made for the CVs.

## 4. Conclusions

1. At low energies (low end of the machine capacity), certain metallic alloys have CVs that are about half that of the plastics in this range. This seems to support the option of using metal impact specimens to verify the performance of plastics impact machines at the low end of their range.
2. Metal specimens would increase the load on the machine striker and frame, permitting better resolution of machine rigidity and mounting problems.
3. Verification testing provides valuable performance data. For example, the data for alloy 6061-T6 indicate that two different designs of impact machines for plastics produce results that agree within the uncertainty that can be attributed to the specimens alone.

## Figures and Tables

**Table 1:** Verification of Charpy impact machine in testing plastics

Specimen ID	Material	Absorbed energy (J)	Notch radius (mm)	Specimen dimensions (mm)	Potential energy (J)	Comments
20	6061-T6	3.576	0.50	4 by 5	5	Remaining ligament
21	6061-T6	3.648	0.50	4 by 5	5	Remaining ligament
22	6061-T6	3.338	0.50	4 by 5	5	Complete breaks
23	6061-T6	3.301	0.50	4 by 5	22	Remaining ligament
24	6061-T6	3.571	0.50	4 by 5	22	Remaining ligament
25	6061-T6	3.550	0.50	4 by 5	22	Complete breaks
26	6061-T6	3.036	0.25	4 by 5	22	Complete breaks
27	6061-T6	3.435	0.25	4 by 5	22	Complete breaks
28	6061-T6	2.858	0.25	4 by 5	22	Complete breaks
29	7075-T6	1.138	0.25	4 by 5	22	Complete laminate breaks
30	7075-T6	1.138	0.25	4 by 5	22	Complete laminate breaks
31	7075-T6	1.239	0.25	4 by 5	22	Complete laminate breaks
32	2219-T87	1.245	0.25	4 by 5	22	Complete brittle fracture
33	2219-T87	1.320	0.25	4 by 5	22	Complete brittle fracture
34	2219-T87	1.382	0.25	4 by 5	22	Complete brittle fracture
35	20l4-T6	2.889	0.25	4 by 5	22	Complete laminated fracture
36	2014-T6	3.000	0.25	4 by 5	22	Complete laminated fracture
37	2014-T6	2.791	0.25	4 by 5	22	Complete laminated fracture
38	2024-T351	1.970	0.25	4 by 5	22	Complete brittle fracture
39	2024-T351	1.829	0.25	4 by 5	22	Complete brittle fracture
40	2024-T351	1.807	0.25	4 by 5	22	Complete brittle fracture
41	606l-T6	3.084	0.50	4 by 5	358	Compare to 26, 27, 28
42	6061-T6	3.084	0.50	4 by 5	358	Compare to 26, 27, 28
43	6061-T6	3.084	0.50	4 by 5	358	Compare to 26, 27, 28
44	606l-T6	3.168	0.25	4 by 5	358	Compare to A, B
C	7075	1.498	0.25	4 by 5	358	
D	7075	1.581	0.25	4 by 5	358	
E	7075	1.831	0.25	4 by 5	358	
F	7075	1.664	0.25	4 by 5	358	
45	4340	2.917	0.25	5 by 5	358	
46	4340	3.168	0.25		358	
47	4340	3.001	0.25	5×5	358	
48	4340	3.001	0.25	5×5	358	
49	4340	1.581	0.25	4 by 4	358	
56-72	4340-LL56	4.552	0.25	4 by 5	22	
56-73	4340-LL56	4.357	0.25	4 by 5	22	
56-74	4340-LL56	4.414	0.25	4 by 5	22	
56-1	4340-LL56	4.386	0.25	4 by 5	22	No side groove
56-2	4340-LL56	4.484	0.25	4 by 5	22	No side groove
56-3	4340-LL56	4.573	0.25	4 by 5	22	No side groove
56-4	4340-LL56	4.430	0.25	4 by 5	22	No side groove
56-5	4340-LL56	4.568	0.25	4 by 5	22	No side groove
56-6	4340-LL56	4.470	0.25	4 by 5	22	No side groove
56-7	4340-LL56	4.382	0.25	4 by 5	22	No side groove
56-8	4340-LL56	4.457	0.25	4 by 5	22	No side groove
56-9	4340-LL56	4.546	0.25	4 by 5	22	No side groove
56-10	4340-LL56	4.298	0.25	4 by 5	22	No side groove
56-31S	4340-LL56	2.363	0.25	4 by 5	22	Side groove
56-32S	4340-LL56	2.467	0.25	4 by 5	22	Side groove
56-33S	4340-LL56	2.495	0.25	4 by 5	22	Side groove
56-34S	4340-LL56	2.519	0.25	4 by 5	22	Side groove
56-35S	4340-LL56	2.535	0.25	4 by 5	22	Side groove
56-36S	4340-LL56	2.483	0.25	4 by 5	22	Side groove
56-37S	4340-LL56	2.447	0.25	4 by 5	22	Side groove
56-38S	4340-LL56	2.535	0.25	4 by 5	22	Side groove
56-39S	4340-LL56	2.447	0.25	4 by 5	22	Side groove
56-40S	4340-LL56	2.404	0.25	4 by 5	22	Side groove
LL11-1	4340	2.888	0.25	4 by 5	22	Machine #2, no side groove
LL11-2	4340	2.739	0.25	4 by 5	22	Machine #2, no side groove
LL11-3	4340	2.725	0.25	4 by 5	22	Machine #2, no side groove
LL11-4	4340	2.793	0.25	4 by 5	22	Machine #2, no side groove
LL11-5	4340	2.725	0.25	4 by 5	22	Machine #2, no side groove
LL11-6	4340	2.685	0.25	4 by 5	22	Machine #2, no side groove
LL11-7	4340	2.698	0.25	4 by 5	22	Machine #2, no side groove
LL11-8	4340	2.671	0.25	4 by 5	22	Machine #2, no side groove
LL11-9	4340	2.671	0.25	4 by 5	22	Machine #2, no side groove
LL11-10	4340	2.752	0.25	4 by 5	22	Machine #2, no side groove
LA46-1	4340	2.210	0.25	4 by 5	22	Machine #2, side groove
LA46-2	4340	2.115	0.25	4 by 5	22	Machine #2, side groove
LA46-3	4340	1.424	0.25	4 by 5	22	Machine #2, side groove
LA46-4	4340	1.356	0.25	4 by 5	22	Machine #2, side groove
LA46-5	4340	1.315	0.25	4 by 5	22	Machine #2, side groove
LA46-6	4340	1.302	0.25	4 by 5	22	Machine #2, side groove
LA46-7	4340	1.342	0.25	4 by 5	22	Machine #2, side groove
LA46-8	4340	1.410	0.25	4 by 5	22	Machine #2, side groove
LA46-9	4340	1.356	0.25	4 by 5	22	Machine #2, side groove
LA46-10	4340	1.397	0.25	4 by 5	22	Machine #2, side groove
G7	2219-T87	0.759	0.25	4 by 5	22	Machine #2, no side groove
G8	2219-T87	0.705	0.25	4 by 5	22	Machine #2, no side groove
G9	2024T-351	1.071	0.25	4 by 5	22	Machine #2, no side groove
G10	2024T-351	1.071	0.25	4 by 5	22	Machine #2, no side groove
G13	2014-T6	2.386	0.25	4 by 5	22	Machine #2, no side groove
G14	2014-T6	1.302	0.25	4 by 5	22	Machine #2, no side groove
G3	7075-T6	1.152	0.25	4 by 5	22	Machine #2, no side groove
G4	7075-T6	0.936	0.25	4 by 5	22	Machine #2, no side groove
G1	6061-T6	2.088	0.50	4 by 5	22	Machine #2, no side groove
G2	6061-T6	1.966	0.50	4 by 5	22	Machine #2, no side groove
G5	6061-T6	1.790	0.50	4 by 5	22	Machine #2, no side groove
G6	6061-T6	2.766	0.50	4 by 5	22	Machine #2, no side groove
G11	7075-T6	0.664	0.25	4 by 5	22	Machine #2, no side groove
G12	7075-T6	0.610	0.25	4 by 5	22	Machine #2, no side groove

**Table 2:** Verification of lzod impact machine in testing plastics

Specimen ID	Material	Absorbed energy (J)	Notch radius (mm)	Specimen dimensions (mm)	Potential energy (J)	Comments
1	4340	1.311	0.25	4 by 4	22	Remaining ligament
2	4340	1.777	0.25	4 by 4	22	Remaining ligament
3	6061-T6	2.743	0.25	4 by 5	22	Remaining ligament
4	6061-T6	2.891	0.25	4 by 5	22	Remaining ligament
5	6061-T6	2.580	0.25	4 by 5	22	Remaining ligament
6	6061-T6	2.817	0.25	4 by 5	22	Remaining ligament
7	6061-T6	2.461	0.25	4 by 5	22	Remaining ligament
A	7075-T6	1.756	0.25	4 by 5	22	Remaining ligament
B	7075-T6	1.390	0.25	4 by 5	22	Remaining ligament
8	4340	1.331	0.25	4 by 4	22	Remaining ligament
9	4340	1.314	0.25	4 by 4	22	Remaining ligament
10	4340	1.314	0.25	4 by 4	22	Remaining ligament
11	4340	2.043	0.25	4 by 5	22	Remaining ligament
12	4340	2.150	0.25	4 by 5	22	Remaining ligament
14	4340	2.125	0.25	4 by 5	22	Remaining ligament
15	4340	2.484	0.25	5 by 5	22	Remaining ligament
16	4340	2.449	0.25	5 by 5	22	Remaining ligament
17	4340	2.557	0.25	5 by 5	22	Remaining ligament
18	4340	2.537	0.25	5 by 5	22	Remaining ligament
19	4340	2.731	0.25	5 by 5	22	Remaining ligament

**Table 3:** Verification of Izod and Charpy impact machines in testing plastics

Specimen ID	Material	Mean absorbed energy (J)	Standard deviation (J)	Coefficient of variation	Notch radius (mm)	Specimen dimension (mm)	Potential energy (J)	Comments
Izod								

1-2, 8-10	4340	1.314	0.111	0.084	0.25	4 by 4		Remaining ligament
3-7	6061-T6	2.698	0.176	0.065	0.50	4 by 5		Remaining ligament
A&B	7075-T6	1.573	0.259	0.165	0.50	4 by 5		Remaining ligament
11-14	4340	2.106	0.558	0.264	0.50	4 by 5		Remaining ligament
15-19	4340	2.511	0.108	0.042	0.50	5 by 5		Remaining ligament

Charpy								

20-22	6061-T6	3.521	0.162	0.046	0.50	4 by 5	5.0	Remaining ligament (20&21)
								Complete break-22
23-25	6061-T6	3.474	0.150	0.043	0.50	4 by 5	22.0	Remaining ligament (23&24)
								Complete break-25
26-28	6061-T6	3.110	0.300	0.097	0.25	4 by 5	22.0	Complete breaks
29-31	7075-T6	1.172	0.058	0.050	0.25	4 by 5	22.0	Complete laminate breaks
32-34	2219-T87	1.316	0.069	0.052	0.25	4 by 5	22.0	Complete brittle fracture
35-37	2014-T6	2.894	0.105	0.036	0.25	4 by 5	22.0	Complete laminated fracture
38-40	2024-T351	1.868	0.088	0.047	0.25	4 by 5	22.0	Complete brittle fracture
41-43	606l-T6	3.084	0.000	0.000	0.50	4 by 5	358	Compare to 26, 27, 28, (44)
44	6061-T6	3.168	0.000	0.000	0.25	4 by 5	358	
C-F	7075	1.644	0.142	0.086	0.25	4 by 5	358	
45-48	4340	3.022	0.105	0.035	0.25	5×5	358	
56-72 to								
56-74	4340-LL56	4.441	0.100	0.023	0.25	4 by 5	22	
56-1 to								
56-10	4340-LL56	4.459	0.089	0.020	0.25	4 by 5	22	No side groove
56-31S to								
56-40S	4340-LL56	2.476	0.068	0.028	0.25	4 by 5	22	Side groove
LL11-1 to								
LL11-10	4340	2.739	0.068	0.025	0.25	4 by 5	22	Machine #2, no side groove
LA46-1 to								
LA46-10	4340	1.519	0.339	0.223	0.25	4 by 5	22	Machine #2, side groove
G7-G8	2219-T87	0.732	0.041	0.056	0.25	4 by 5	22	Machine #2, no side groove
G9-G10	202T4-351	1.071	0.000	0.000	0.25	4 by 5	22	Machine #2, no side groove
G13-G14	2014-T6	1.844	0.732	0.397	0.25	4 by 5	22	Machine #2, no side groove
G3-G4	7075-T6	1.044	0.149	0.143	0.50	4 by 5	22	Machine #2, no side groove
G1-G2	6061-T6	2.027	0.081	0.040	0.50	4 by 5	22	Machine #2, no side groove
G5-G6	6061-T6	2.278	0.691	0.304	0.25	4 by 5	22	Machine #2, no side groove
G11-G12	7075-T6	0.637	0.041	0.064	0.25	4 by 5	22	Machine #2, no side groove

**Table 4:** Effect of alloy^a^

Material	Number of specimens	Mean absorbed energy (J)	Standard deviation (J)	Coefficient of variation
4340	3	4.441	0.100	0.023
2014-T6	3	2.894	0.105	0.036
2024-T351	3	1.868	0.088	0.047
2219-T87	3	1.316	0.069	0.052
6061-T6	3	3.110	0.300	0.097
7075-T6	3	1.172	0.058	0.050

a Notch radius 0.25 mm. Specimen dimension 4 mm by 5mm. Potential energy 22 J.

**Table 5:** Effect of side grooves^a^

Material	Number of specimens	Mean absorbed energy (J)	Standard deviation (J)	Coefficient of variation	Side grooves
4340	10	4.459	0.089	0.020	N
4340	10	2.476	0.068	0.028	Y

a Notch radius 0.25 mm. Specimen dimension 4 mm by 5 mm. Potential energy 22 J.

**Table 6:** Effect of notch radius^a^

Material	Number of specimens	Mean absorbed energy (J)	Standard deviation (J)	Coefficient of variation	Side grooves
6061-T6	3	3.474	0.150	0.043	0.50
6061-T6	3	3.110	0.300	0.096	0.25

a Specimen dimension 4 mm by 5 mm. Potential energy 22 J.

**Table 7:** Effect of machine capacity^a^

Material	Number of specimens	Mean absorbed energy (J)	Standard deviation (J)	Coefficient of variation	Notch radius (mm)	Machine potential energy (J)
6061-T6	3	3.521	0.162	0.046	0.50	5
6061-T6	3	3.474	0.150	0.043	0.50	22
6061-T6	3	3.110	0.300	0.097	0.25	22
6061-T6	3	3.084	0.000	0.000	0.50	358
6061-T6	1	3.168	0.000	0.000	0.25	358
7075-T6	4	1.644	0.142	0.086	0.25	358
7075-T6	3	1.172	0.058	0.050	0.25	22

a Specimen dimension 4 mm by 5 mm. Potential energy 22 J.
